# Inhibition of Extracellular Matrix Protein Fibulin-3 Reduces Immunosuppressive Signaling and Increases Macrophage Activation in Glioblastoma

**DOI:** 10.1158/2767-9764.CRC-25-0083

**Published:** 2025-09-11

**Authors:** Somanath Kundu, Soham Mitra, Arivazhagan Roshini, John A. Longo, Sharon L. Longo, Abigail G. Venskus, Harish Babu, Mariano S. Viapiano

**Affiliations:** 1 Department of Neuroscience and Physiology, State University of New York - Upstate Medical University, Syracuse, New York.; 2 Department of Neurosurgery, State University of New York - Upstate Medical University, Syracuse, New York.

## Abstract

Glioblastoma (GBM) tumors remain a challenge for immunotherapy owing to their heterogeneous and immunologically cold properties. GBM cells change the composition of the neural extracellular matrix (ECM), affecting the mobility, survival, and function of immune cells such as tumor-associated microglia and infiltrated macrophages (TAM). The ECM protein fibulin-3/*EFEMP1* is a pericellular component uniquely upregulated in GBM compared with the normal brain, which promotes tumor growth and invasion. In this study, we demonstrate that fibulin-3 indirectly modulates TAM behavior and can be targeted to restore innate immune responses. Intracranial tumors initiated by fibulin-3–deficient GBM cells showed increased presence of TAMs with decreased immunosuppression markers (arginase-1, CD206), whereas the opposite effects were observed in tumors overexpressing fibulin-3. *In silico* analyses revealed a positive correlation between fibulin-3 and an immunosuppressive signature that was validated in GBM stem cells and *in vivo*. We further demonstrated that fibulin-3 regulates the expression of immunosuppressive signals (CSF-1, TGF-β1, and CD47) by autocrine activation of NF-κB signaling. Immunosuppressive signals were downregulated by knockdown of fibulin-3 in GBM stem cells and by inhibition of this protein using an anti–fibulin-3 antibody. Myeloid cells exhibited higher phagocytic activity and killing of GBM cells in presence of this antibody. Furthermore, locoregional delivery of anti–fibulin-3 in mice with intracranial GBM increased the presence of proinflammatory TAMs, thereby reducing tumor viability. Our findings show that anti–fibulin-3 approaches, which affect the ECM surrounding tumor and immune cells, can diminish immunosuppression in GBM and boost innate immune responses against the tumor.

**Significance:**

Inhibition of the ECM protein fibulin-3, which is highly upregulated in glioblastoma tumors, decreases immunosuppressive signals produced by the tumor cells and exposes them to increased attack by TAMs.

## Introduction

Advances in cancer immunotherapy have raised hope for improved treatment of malignant gliomas, including glioblastoma (GBM), which is the most common and aggressive form of primary malignant brain cancer (1). However, malignant gliomas remain a formidable challenge for immune-based treatments because of their molecular heterogeneity, poor immunogenicity, and growth in the largely isolated and immunosuppressive neural environment (2–4).

High-grade gliomas are densely populated by local microglia and infiltrated bone marrow (BM)-derived macrophages (5, 6), which can make up to a third of the total cells recovered from GBM specimens (7). These myeloid cells are attracted to the tumor mass by tissue damage and the wound-like properties of the tumor (8) that elicit inflammation. Tumor-associated macrophages and microglia (TAM) are then co-opted by immunosuppressive signals from the tumor, inhibiting further immune responses and unwittingly supporting tumor growth. The resulting “tumor-promoting” transcriptional and phenotypic changes in TAMs have been likened to the M2-like polarized phenotype of macrophages treated *in vitro* with immunosuppressive cytokines (9), although it is well accepted that the phenotypes of TAMs are plastic and heterogeneous and cannot be ascribed to a simple snapshot of gene expression (10). Nevertheless, it is clear that TAMs have a distinct tumor-promoting role and that, to succeed, GBM immunotherapies must reduce the presence of these cells in the tumor or revert them to an antitumor inflammatory status (11). Remarkably, spatial changes in TAM polarization from the tumor periphery (proinflammatory) toward the core (immunosuppressive) correlate with changes in extracellular matrix (ECM) components (12–14), highlighting the potential involvement of the ECM in GBM immunosuppression.

The ECM of gliomas is a complex and unique scaffold surrounding malignant and tumor-associated cells, composed of hyaluronic acid, central nervous system (CNS)-specific glycoproteins secreted mainly by astrocytes, and a host of soluble factors and proteins produced by the tumor cells, many of which are absent from the normal brain ECM (15). Several ECM components are important regulators of immune cell behavior in GBM (16–18). For example, hyaluronic acid and ECM proteins such as osteopontin or tenascins can limit the infiltration of T cells into GBM (19, 20) and regulate myeloid cell migration, differentiation, and polarization in these tumors (21, 22). Nevertheless, the possibility that tumor-secreted ECM molecules act as inducers of immunosuppression in GBM remains largely unexplored and therapeutically unexploited.

Fibulin-3 (*EFEMP1*) is a pericellular (surface-associated) ECM protein secreted by GBM cells, common to all GBM subtypes but absent from the normal brain (23). Fibulin-3 has multiple tumor-promoting functions in GBM and other solid tumors, such as increasing tumor cell proliferation and invasion, chemoresistance, and the survival of the tumor stem cell population (24, 25). Fibulin-3 can be inhibited with a function-blocking antibody, mAb428.2, which prevents this protein from activating NF-κB signaling in GBM and PI3K/Akt signaling in mesothelioma, resulting in the extended survival of mice carrying fibulin-3–expressing tumors (26, 27). Interestingly, this antibody has modest effects against GBM cells cultured alone compared with its more evident apoptotic, antiangiogenic, and pronecrotic effects in the tumor mass (26), suggesting that the antibody triggers a stronger than expected antitumor response *in vivo*. Indeed, mAb428.2 treatment of mice carrying subcutaneous, fibulin-3–expressing tumors increases the infiltration of macrophages expressing proinflammatory cytokines (26) that may act against the tumor. However, whether fibulin-3 affects the behavior of TAMs in cancer has not been investigated.

In this study, we demonstrate that fibulin-3 has a previously undescribed immunomodulatory role in GBM and regulates the expression of myeloid immunosuppresive molecules in GBM cells. We further show that targeted knockdown of fibulin-3 or inhibition of this protein with a novel humanized fibulin-3–targeting antibody reduces the immunosuppressed status of TAMs and increases macrophage cytotoxicity against GBM cells.

## Materials and Methods

### Cells and biological reagents

Fresh surgical specimens were procured after obtaining written informed consent from patients with GBM following recognized ethical guidelines (US Common Rule) and approval by SUNY Upstate Medical University Institutional Review Board. GBM stem cell (GSC) cultures established from these specimens have been previously described (26) and were authenticated using the “Cell Check” service provided by IDEXX-Research Animal Diagnostic Laboratory. GSCs chosen for this study have a representative range of expression of fibulin-3 that correlates with their expression of immunosuppressive signals (Supplementary Fig. S1). U251MG (human, RRID: CVCL_0021), GL261 (mouse, RRID: CVCL_Y003), and CNS1 (rat, RRID: CVCL_5276) GBM cells were cultured as described previously (23). U937 (RRID: CVCL_0007) and THP-1 (RRID: CVCL_0006) myeloid cells were cultured in RPMI-1640 (Corning) supplemented with 300 mg/L L-glutamine and 10% FBS and then differentiated into macrophages using 100 ng/mL phorbol 12-myristate 13-acetate (Sigma-Aldrich, #P8139) for 24 hours (THP-1) or 48 hours (U937). Human peripheral blood mononuclear cells were isolated from whole blood (New York Blood Center) using density gradient centrifugation in Lymphoprep (Stemcell Technologies) and cultured in Iscove’s modified Dulbecco’s medium (Corning) supplemented with 9% FBS and 1% human serum. Mouse myeloid cells were isolated from the bone marrow of C57Bl/6 adult mice and cultured in RPMI-1640 medium containing 300 mg/mL glutamine and 10% FBS. Human and mouse monocytes were differentiated into mature macrophages using 50 ng/mL M-CSF (Peprotech, #300-25) and 25 ng/mL GM-CSF (Peprotech, #300-03) for 6 days. HMC3 microglia cells (RRID: CVCL_II76) were cultured in DMEM (Corning) containing 10% FBS. Cocultures were established in Opti-MEM medium (Invitrogen). All cultures contained standard penicillin/streptomycin. All cells were tested for absence of *Mycoplasma* contamination by qRT-PCR on a monthly basis and were used within 10 passages after recovery from their initial frozen stocks.

Fibulin-3 cDNA and RNAi sequences have been described and validated previously (28). The constructs were introduced into GBM cells by lentiviral transduction (28). All GBM cells were transduced with the lentiviral vectors pCDH-EF1-copGFP (System Biosciences, RRID: Addgene_73030) or LPP-hLUC-LV206 (GeneCopoeia) for the expression of fluorescent or luminescent markers, respectively.

The monoclonal anti–fibulin-3 antibody, mAb428.2, has been described and validated for its ability to inhibit fibulin-3 function (26). In this study, a fully humanized variant of mAb428.2 was developed by modifying the original mouse V_H_ and V_L_ sequences and inserting them into a human IgG1 backbone using the Prometheus process (Absolute Antibody Ltd.; ref. 29). Affinity-purified humanized mAb428.2 was validated by ELISA, Western blotting, and *in vitro* assays (Supplementary Fig. S1) and compared against the predecessor chimeric mAb428.2 (mouse V_H_/V_L_ in a human IgG1 backbone) previously reported (27). Additional reagents used in this study included purified fibulin-3 (R&D Systems, # 8416-FB), purified human IgG1 and human IgG Fc fragment (Rockland Immunochemicals), and the NF-κB inhibitor caffeic acid phenetyl-ester (CAPE, Cayman Chemical, #70750). Commercially available antibodies and PCR primers used in this study are listed in Supplementary Tables SI and SII, respectively.

### *In vitro* assays

To measure gene and protein expression, cultured cells were dissociated mechanically without enzymatic treatment and processed for semi-quantitative RT-PCR or Western blotting following standard procedures. RNA was collected from transfected cells using Pure Link RNA Mini Kit (Invitrogen, #12183018A). Lipofectamine-mediated transfection for transient gene overexpression or knockdown was performed 24 to 48 hours before cell collection. Macrophages or microglia, derived from cell lines or primary cells, were cocultured with GBM cells in 96-well plates at an effector:target ratio of 5:1 (2.5 × 10^4^ macrophages/well) for 48 hours to assess GBM cytotoxicity and at an effector:target ratio of 1:1 (5 × 10^5^ macrophages/well) for 60 minutes to visualize phagocytic activity. Cytotoxicity was quantified by a decrease in the fLuc signal of the tumor cells (BrightGlo Luc Kit, Promega, #E2610) and further validated by measuring the released lactate dehydrogenase enzyme as a separate measure of cell death (CytotTox Kit, Promega, #G1780). To quantify the phagocytosis of GFP-expressing GSCs, cocultures were lightly fixed with 4% phosphate-buffered paraformaldehyde and prepared for flow cytometry following standard procedures, using CD45 to gate the macrophage population. In parallel experiments, nonfluorescent GSCs were briefly incubated with a pH-sensitive dye (pHrodo Green; Molecular Probes, #P35369) before coculture, and the uptake of tumor cell fragments in macrophage lysosomes was confirmed by flow cytometry. Some cocultures were processed for confocal microscopy to directly visualize GSC-derived fragments inside the macrophages.

### *In vivo* studies

All animal experiments were performed following Institutional Animal Care and Use Committee approval at SUNY Upstate Medical University. GSCs (2 μL containing 1 × 10^4^ cells/μL) were implanted in the right striatum of 8-week-old athymic nude mice (FoxN1^nu/nu^, 1:1 female:male ratio, The Jackson Laboratory, RRID: IMSR_JAX:007850) as previously described (28). GL261 (2 μL containing 5 × 10^4^ cells/μL) and CNS1 cells (3 μL containing 5 × 10^4^ cells/μL) were implanted in the same brain region of 8-week old C57Bl/6 mice (RRID: IMSR_JAX:000664) and Lewis rats (RRID: RGD_737932; ref. 23), respectively. To induce the expression of fibulin-3 short hairpin RNAs, mice received doxycycline (1 mg/mL, MP Biomedicals, #02198955) in drinking water containing 2% w/v sucrose and were allowed to drink medicated water *ad libitum* after surgery. Fibulin-3 cDNA was constitutively overexpressed and did not require induction. Tumors were allowed to grow for 14 to 21 days, after which the animals were euthanized, perfused with PBS and paraformaldehyde, and had their brains processed for IHC. Some tumors were freshly resected at an earlier time point (8–10 days after implantation, to avoid their hemorrhagic stage), dissociated mechanically and enzymatically (1 mg/mL type-IV collagenase and 0.25 mg/mL DNAse I, Worthington Biochemical), and fractionated in a 30/70% Percoll gradient (Cytiva). The enriched mononuclear immune cell fraction was processed for semi-quantitative RT-PCR or flow cytometry using an LSRFortessa cell analyzer (Becton Dickinson). Cytometric results were analyzed using FlowJo v.10.1 (RRID: SCR_008520).

To treat intracranial tumors with humanized anti–fibulin-3 Ab428.2 or control IgG1 antibodies, animals were operated a week after tumor injection to implant an intracranial cannula and a subcutaneous osmotic reservoir (Alzet, #2001 containing 5 mg/mL antibody), as described previously (26). The animals received an antibody infusion for 8 days and were euthanized 48 hours after completing the antibody treatment.

### External datasets and data analysis

Expression of fibulin-3 and immunomodulatory genes was extracted from the IDH1 wild-type GBM datasets available in The Cancer Genome Atlas (RRID: SCR_003193) and Chinese Glioma Genome Atlas (RRID: SCR_018802) databases, accessed using the aggregator website GlioVis (30). The bulk transcriptional subtypes (proneural, mesenchymal, and classic) of GBM were identified using an approach proposed by Wang and colleagues (31). Genes with direct transcriptional regulation by NF-κB were compiled from previously reported datasets (32).

For RNA sequencing of transfected cells, libraries were prepared from poly A–enriched RNA and sequenced on an Illumina HiSeq instrument with 2 × 150 bp paired-end reads and an average coverage depth of >25 million reads/sample. Sequencing reads were mapped to the hg38 human genome and quantified using Partek Flow software (RRID: SCR_011860). Gene counts were processed to remove cell line–dependent batch effects and quantified as trimmed mean of M-values for analysis. Gene ontology was analyzed using the Gene Set Enrichment Analysis tool provided by Partek Flow.

All *in vitro* experiments were performed in triplicate, with three independent replicates per condition. The *in vivo* studies used *N* = 5–8 per condition. IHC quantification was performed by investigators blinded to the experimental conditions using at least five separate tissue sections per animal. Image analysis was performed using *ad hoc* scripts in ImageJ (v. 1.54, RRID: SCR_003070) to quantify IBA1-positive, CD206-positive, and colocalized IBA1/CD206 pixels in each tumor. Values in the graphs represent the mean ± SD. Comparisons between two conditions were analyzed using Student t test or multiple *t* tests with multiple comparison correction. Grouped results were analyzed using one- or two-way ANOVA, depending on the experimental design. Differences were considered statistically significant at *P* < 0.05. Statistical differences in figure panels are represented as *, < 0.05; **, < 0.01; ***, < 0.001, and ***, < 0.0001.

### Data availability

RNA sequencing data produced in this study are publicly available in Gene Expression Omnibus at GSE284269. Additional data generated in this study are available upon request from the corresponding author.

## Results

### Fibulin-3 expression by GBM cells correlates with infiltration of tumor-promoting TAMs

**Figure 1.**
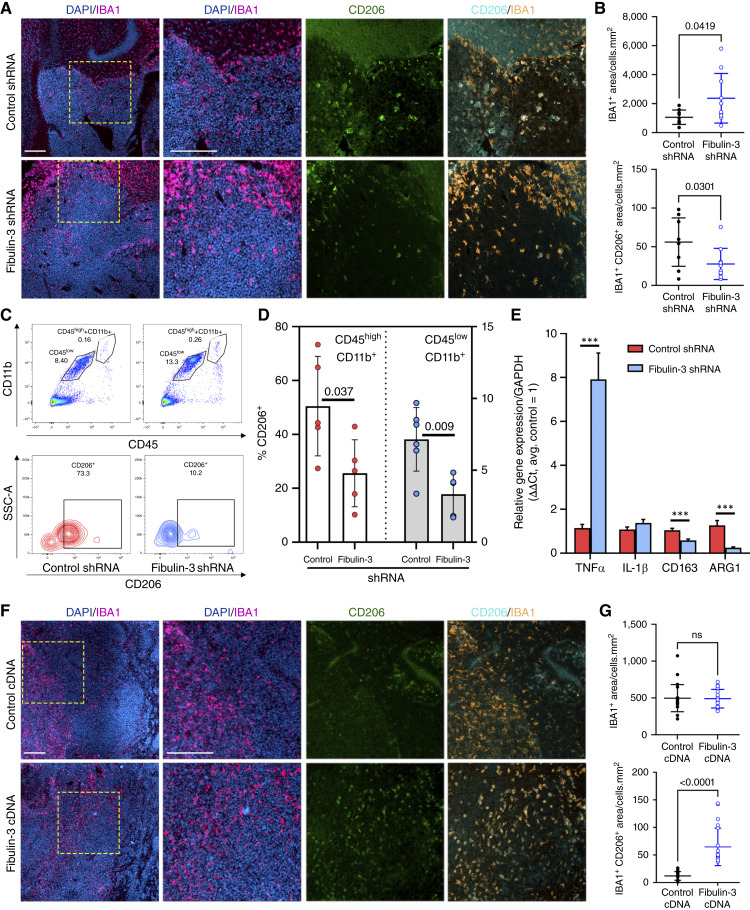
Fibulin-3 expression affects the accumulation and phenotype of TAMs. **A,** Representative images of GSC-derived intracranial tumors (GBM08 line) expressing control or fibulin-3 short hairpin RNAs (shRNA), showing TAM density (IBA1 staining) and expression of the immunosuppression marker CD206 in TAMs; cell nuclei were stained with 4′,6-diamidino-2-phenylindole (DAPI). Scale bars, 200 μm. **B,** Quantification of total TAMs (IBA1^+^ area within the tumor) and IBA1/CD206 coexpression in TAMs. The results were analyzed by Welch’s corrected *t* test. **C,** Representative flow cytometry gating of TAM populations (macrophages: CD45^hi^ CD11b^+^; microglia: CD45^lo^ CD11b^+^) recovered from the tumors indicated in **A**; note that the peripheral macrophage population is small due to the tumors being resected at an early stage. **D,** Flow cytometry quantification of CD206^+^ in TAMs recovered from freshly resected tumors; comparison by Student *t* test. **E,** qRT-PCR for selected genes expressed in TAMs recovered from control and fibulin-3–deficient tumors; analysis by multiple paired *t* tests with correction for multiple comparisons. **F,** Representative images of CNS1-derived intracranial tumors expressing control or fibulin-3 cDNA; stains are the same as in **A**; scale bars, 200 μm. **G,** Quantitative analysis of the tumors in (**F**) showing total TAMs (IBA1^+^ area within the tumor) and IBA1/CD206 coexpression in TAMs. The results were analyzed by Welch’s corrected *t* test. ns, not significant.

Fibulin-3 enhances GBM invasion (23), stemness (28, 33), vascularization (34), and peritumoral astrocytosis (32); however, the effects of this protein on immune cells in GBM remain unexplored. To address this question, we first analyzed the presence of TAMs in control and fibulin-3–deficient proneural GSC-derived tumors (GBM08 cells) implanted in athymic mice. Our results showed a significant increase of IBA1-positive TAMs in fibulin-3–deficient tumors compared with controls (Fig. 1A and B). Simultaneously, we observed a significant decrease in expression of the immunosuppression marker CD206 in these myeloid cells. These results were confirmed using a second, more aggressive mesenchymal GSC model (GBM34 cells, Supplementary Fig. S2). To avoid quantification bias due to the proapoptotic effects of fibulin-3 knockdown in tumor cells, we chose tissue sections of similar cross-sectional area and cellular density in all animals (quantified in Supplementary Fig. S2). Athymic mice are thought to retain functional myeloid cells (35), although the absence of mature T cells in these animals is an intrinsic weakness for studies involving human xenografts. Accordingly, we further validated our observations in an immunocompetent model using control and fibulin-3–deficient CNS1 cells implanted in Lewis rats (Supplementary Fig. S2). Our results for CNS1-derived tumors were essentially the same as those observed for GSCs.

To confirm our quantitative IHC results, we isolated TAMs from freshly resected control and fibulin-3–deficient GBM34 tumors and quantified CD206 expression in the CD45^hi^/CD11b^+^ (macrophages) and CD45^lo^/CD11b^+^ (microglia) populations. We observed a significant decrease in CD206 expression in both TAM populations isolated from fibulin-3–deficient tumors (Fig. 1C and D). Furthermore, analysis of these TAMs by qRT-PCR showed increased expression of a proinflammatory marker (TFN-α) and reduced expression of genes associated with the immunosuppressed phenotype (CD163 and arginase-1, Fig. 1E).

Next, we performed similar IHC quantification of tumors overexpressing fibulin-3. We chose two cell models (CNS1 and GL261) that have moderate expression of fibulin-3 and are suitable for gain-of-function through overexpression of this protein (23). The results with CNS1 cells implanted in Lewis rats did not show significant changes in the number of IBA1-positive cells but demonstrated a significant increase in CD206 expression in these TAMs (Fig. 1F and G). The results for GL261 cells implanted into C57Bl/6 mice (Supplementary Fig. S2) revealed a modest decrease in TAM infiltration and a large increase in CD206 expression.

Taken together, these results strongly suggest that high expression of fibulin-3 by GBM cells correlates with immunosuppressed features of TAMs, whereas decreased expression of fibulin-3 correlates with increased infiltration of TAMs having a less immunosuppressed and more inflammatory-prone profile. Accordingly, we investigated whether fibulin-3 could function as an immunosuppressive factor in GBM.

### Fibulin-3 correlates with an immunosuppressive signature in GBM tumors and GSCs

**Figure 2.**
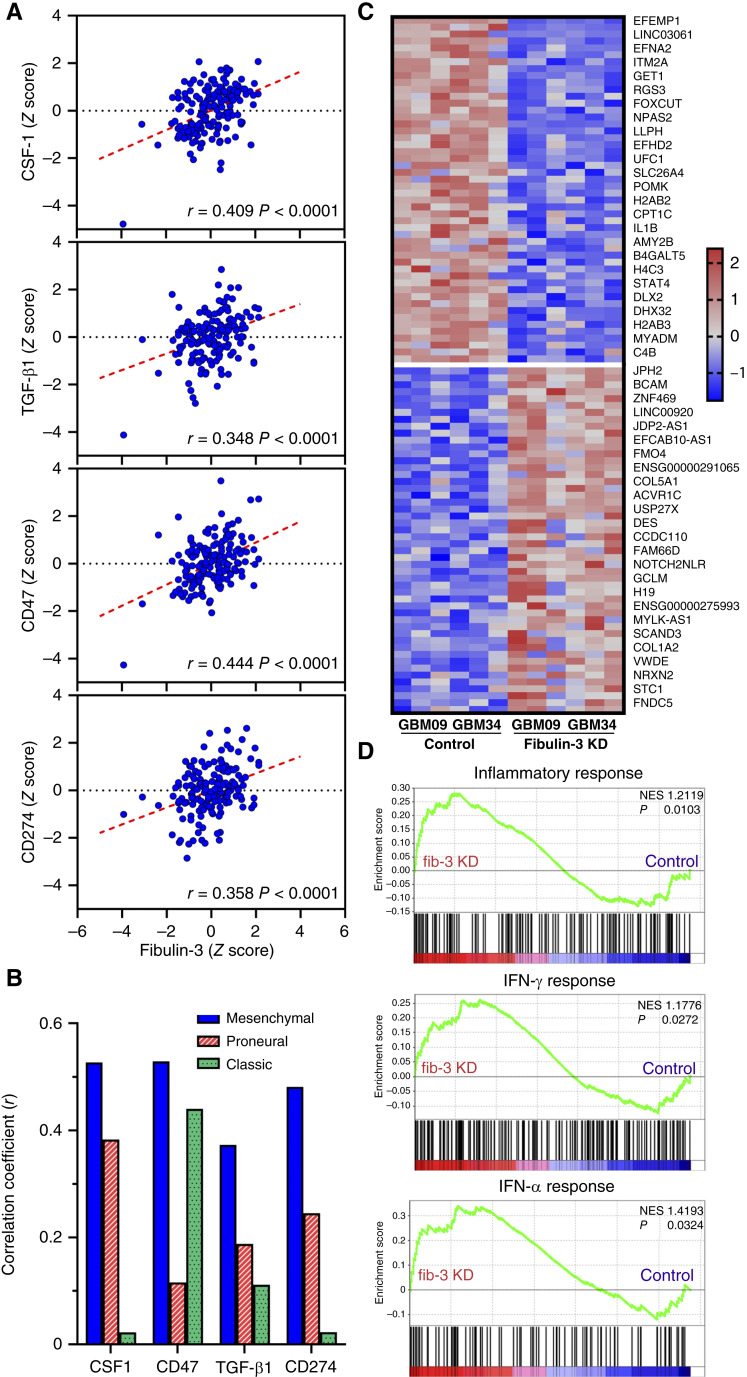
Fibulin-3 expression correlates with an immunosuppressive signature in GBM. **A,** Correlation of fibulin-3 mRNA expression with the expression of CSF-1, TGF-β1, and the checkpoints CD47 and CD274 (PD-L1), extracted from The Cancer Genome Atlas GBM dataset. **B,** Correlation of fibulin-3 with the markers indicated in (**A**) in GBM tumors separated by their bulk transcriptional subtype. **C,** Heatmap showing top gene expression changes in two GSC lines (GBM34 and GBM09) stably transfected with control or fibulin-3 short hairpin RNAs. **D,** Changes in gene expression observed in hallmark signatures for inflammatory response and signaling by IFN-γ and -α (data analyzed using GSEA v.4.3.3), comparing fibulin-3–deficient GSCs (*fib-3 KD*) vs. control cells. KD, knockdown; NES, normalized enrichment score.

Fibulin-3 activates NF-κB signaling in GBM cells and correlates with the expression of numerous NF-κB–regulated genes (32). Several immunomodulatory signals have been identified as direct targets of NF-κB regulation at the transcriptional level (compiled list at http://www.bu.edu/NF-kB/gene-resources/target-genes), including the cytokines CSF-1, TGF-β1, and IL-10 as well as the checkpoint inhibitors CD274 (PD-L1), CD80, and CD47, all of which form an immunosuppressive signature. We analyzed the expression of these genes in clinical GBM specimens using The Cancer Genome Atlas and Chinese Glioma Genome Atlas datasets and found a significant positive correlation between fibulin-3 and this immunosuppressive signature (Fig. 2A; Supplementary Fig. S3). As expected, the cumulative correlation between fibulin-3 and immunosuppressive signals was the highest in mesenchymal tumors (Fig. 2B), which had the highest expression of fibulin-3 (28) and a predominant immune component (31).

We validated these findings by knocking down fibulin-3 in two mesenchymal GSC models with high endogenous fibulin-3 expression (GBM09 and GBM34) and analyzing the resulting transcriptomic changes in these cells (Fig. 2C). Although genes associated with immunomodulation were not among the top genes showing changes after fibulin-3 knockdown (as expected, given other predominant functions of fibulin-3 on tumor progression), gene ontology analysis revealed increased expression of genes associated with inflammatory, IFN-γ, and IFN-α responses (Fig. 2D), suggesting that fibulin-3 downregulates these genes in GSCs.

**Figure 3.**
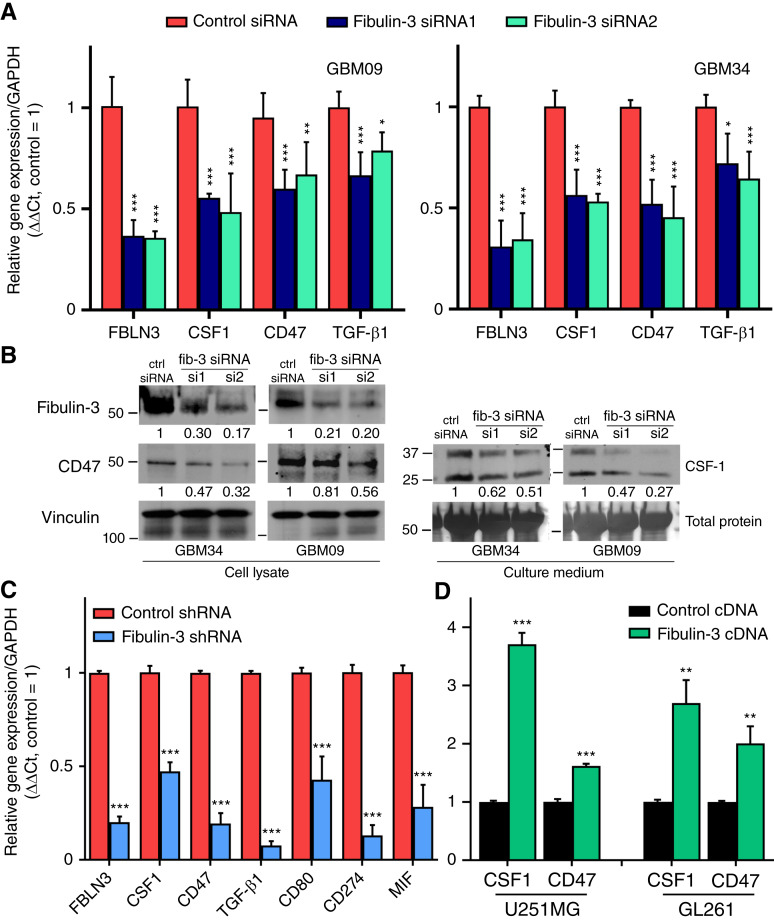
Fibulin-3 regulates the expression of immunosuppressive signals in GBM cells. **A,** Transient transfection of fibulin-3 siRNAs in GSCs results in decreased expression of CSF-1, TGF-β1, and the innate immune checkpoint CD47. **B,** Same results as in (**A**), validated by Western blotting. Total protein loading (amido black stain of blotting membranes) was used as a loading control to normalize the expression of proteins detected in culture medium. The numbers indicate relative integrated optical density for each band, normalized to the loading control and to total protein measurement. ctrl, control; fib-3, fibulin-3. **C,** qRT-PCR of human fibulin-3 (*FBLN3*) and immunosuppressive signals detected in intracranial GSC-derived tumors expressing control or fibulin-3 short hairpin RNAs (shRNA; human-specific primers were used to detect human mRNA in tissue resected from mouse brains). Statistical analysis performed by multiple paired *t* tests with multiple comparison correction. **D,** Expression of CSF-1 and CD47 in the GBM cell lines U251MG and GL261, transfected to overexpress fibulin-3. Statistical analysis by one-way ANOVA for each cell line.

To further confirm an immunosuppressive function of fibulin-3 in GSCs, we investigated changes in genes that affect TAM phenotype. Knockdown of fibulin-3 in GSCs decreased the expression of the cytokines CSF-1 and TGF-β1, as well as the cell surface receptor CD47, which inhibits phagocytosis and acts as an innate immune checkpoint (Fig. 3A and B). Interestingly, other innate immune checkpoints such as CD24 and B2M were not affected (Supplementary Fig. S4). We then returned to the intracranial GSC-derived fibulin-3–deficient tumors (Fig. 1A–E) and performed qRT-PCR for human-specific transcripts. In agreement with our previous results, we observed that fibulin-3–deficient tumors had lower expression of multiple immunosuppressive signals compared with controls (Fig. 3C), aligning with the increased presence of TAMs with low CD206 expression. Finally, we transiently overexpressed fibulin-3 cDNA in GBM cell lines (U251MG and GL261, Fig. 3D) as well as in a colorectal cancer cell line that lacks endogenous fibulin-3 expression [Colo201, RRID: CVCL_F399 (26), Supplementary Fig. S5]. Forced overexpression of fibulin-3 significantly increased the expression of CSF-1 and CD47 in all these cells.

### Fibulin-3 drives the expression of immunosuppressive signals in GBM cells via NF-κB signaling

**Figure 4.**
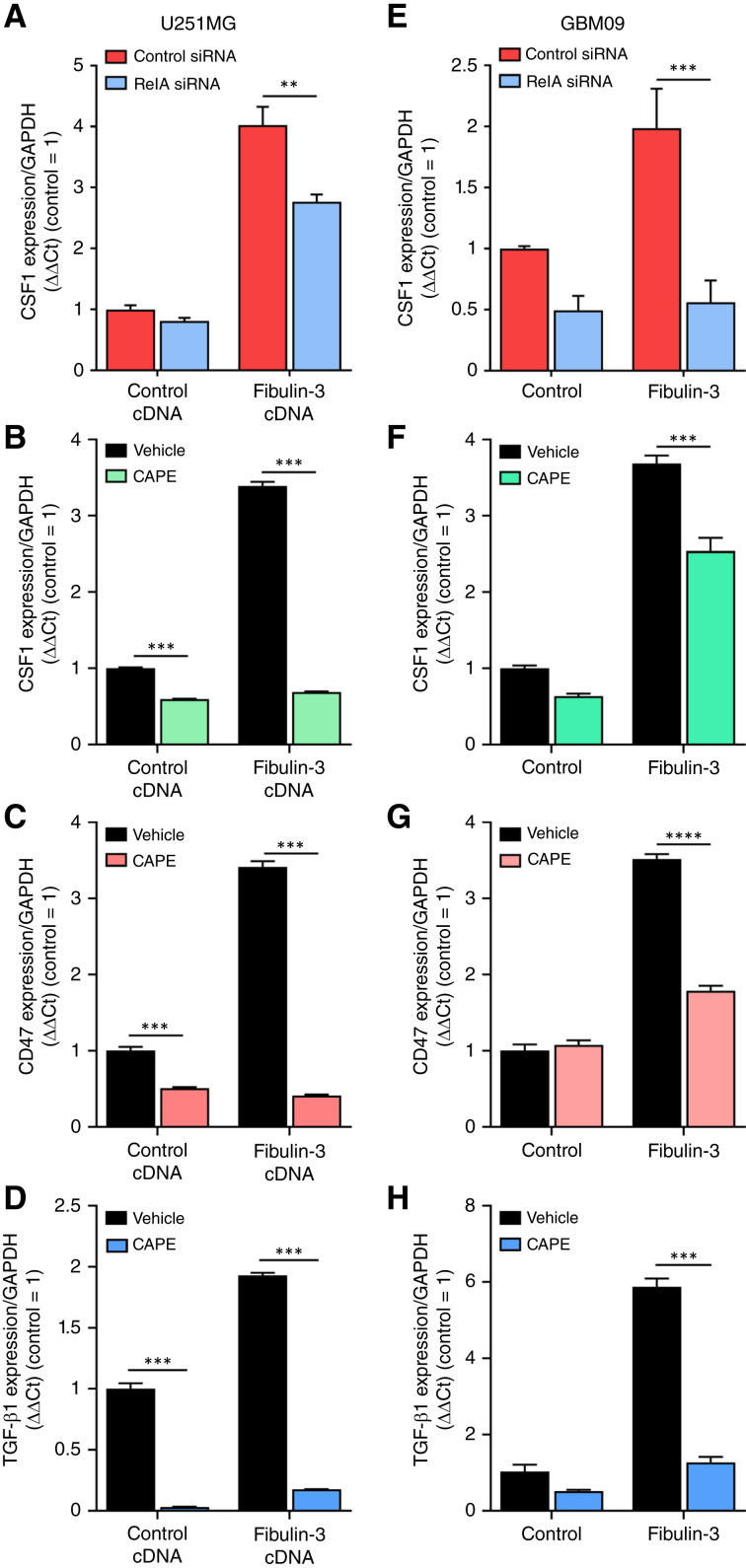
Fibulin-3 regulates the expression of immunomodulatory signals in GBM cells via NF-κB signaling. **A,** Expression of CSF-1 mRNA in U251MG cells cotransfected with fibulin-3 cDNA and p65/RelA siRNA, or their respective controls. **B–D,** Expression of CSF-1 (**B**), CD47 (**C**), and TGF-β1 (**D**) in control and fibulin-3–overexpressing U251MG cells in presence of the NF-κB inhibitor, CAPE (20 μmol/L), or its vehicle. **E,** Expression of CSF-1 mRNA in GBM9 GSCs transfected with control or p65/RelA siRNAs and exposed overnight to purified fibulin-3 (500 ng/mL). **F–H,** Expression of CSF-1 (**F**), CD47 (**G**), and TGF-β1 (**H**) in GBM9 cells exposed to purified fibulin-3, CAPE, or their respective vehicles. The results in all panels were analyzed by two-way ANOVA.

Our results strongly suggested that fibulin-3 induces the coordinated expression of immunosuppressive signals in GBM cells, affecting the phenotype of local and infiltrating TAMs. We further explored the mechanism by which fibulin-3 regulates these signals in a conventional cell line (U251MG) transfected with fibulin-3 cDNA and a mesenchymal GSC line (GBM09) exposed to purified fibulin-3 (500 ng/mL). Overexpression of fibulin-3 in U251MG cells significantly increased the expression of CSF-1 mRNA, as expected (Fig. 4A and B). This effect was largely diminished by knockdown of the canonical NF-κB transcription factor p65/RelA [following a previously validated procedure (32)] and abolished by treating the cells with the low-toxicity NF-κB inhibitor CAPE (20 μmol/L). NF-κB inhibition also inhibited the increased expression of CD47 and TGF-β1 by fibulin-3 in U251MG cells (Fig. 4C and D). Similar results were observed in GBM09 cells; the enhancing effect of purified fibulin-3 on the expression of CSF-1, CD47, and TGF-β1 was significantly diminished by p65/RelA knockdown (Fig. 4E) or CAPE treatment (Fig. 4F–H). We further confirmed that exposure of GBM09 cells to exogenous fibulin-3 increased the binding of p65/RelA to the promoter of *CSF1* gene (Supplementary Fig. S6). Together, these results indicate that fibulin-3 promotes an immunosuppressive signature in GBM cells via NF-κB–mediated transcriptional regulation.

### Blockade of fibulin-3 increases the presence of inflammatory macrophages that target GBM cells

Next, we investigated whether inhibition of fibulin-3 after tumor formation was sufficient to revert the immunosuppressed phenotype of TAMs. We implanted intracranial tumors (mesenchymal GBM09 cells) in athymic mice and, once the tumors were established, we locally infused humanized anti–fibulin-3 mAb428.2 (26) or a control IgG1. Tumors were processed shortly after completing the antibody infusion and analyzed by quantitative IHC for IBA1 and colocalized IBA1/CD206, as indicated in Fig. 1 (the results were again normalized to the tumor cross-sectional area and total cell density, Supplementary Fig. S2).

**Figure 5.**
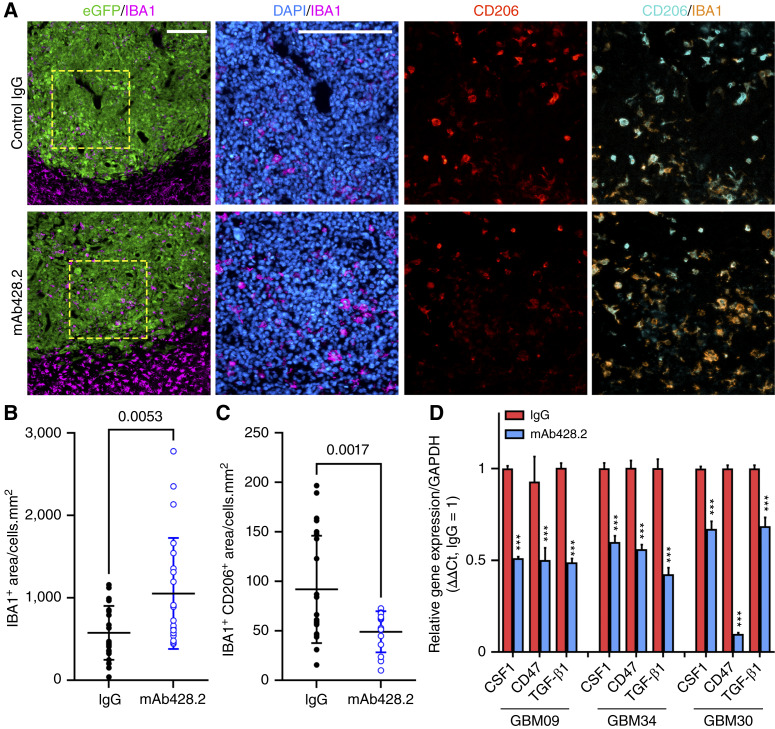
Anti–fibulin-3 antibody increases intratumoral presence of proinflammatory TAMs. **A,** Representative images of intracranial GSC-derived tumors (GBM09) treated with humanized anti–fibulin-3 mAb428.2 or its IgG control. Tissues were stained to reveal TAM density (IBA1) and expression of CD206 in TAMs. Cell nuclei were stained with 4′,6-diamidino-2-phenylindole (DAPI). Scale bars, 200 μm. **B** and **C,** Quantification of total TAMs (**B**) and CD206 expression in TAMs (**C**) in tumors treated with mAb428.2 or its control; data were analyzed by Welch’s corrected *t* test. **D,** Expression of CSF-1, TGF-β1, and CD47 in three different GSC cultures treated with mAb428.2 or its isotype control. Analysis was done by multiple paired *t* tests for each GSC line.

Tumor treatment with mAb428.2 resulted in increased accumulation of TAMs with lower expression of CD206 compared with controls (Fig. 5A–C). In agreement, qRT-PCR of resected tumor tissue using mouse-specific primers demonstrated a significant decrease in immunosuppressive markers and a trend toward upregulation of proinflammatory markers in tumors treated with anti–fibulin-3 antibody (Supplementary Fig. S7). Together, these results closely matched our results observed with fibulin-3 knockdown (Fig. 1). Furthermore, treatment of fibulin-3–expressing GSC lines with mAb428.2 (50 μg/mL) significantly decreased their expression of CSF-1, TGF-β1, and CD47 (Fig. 5D).

**Figure 6.**
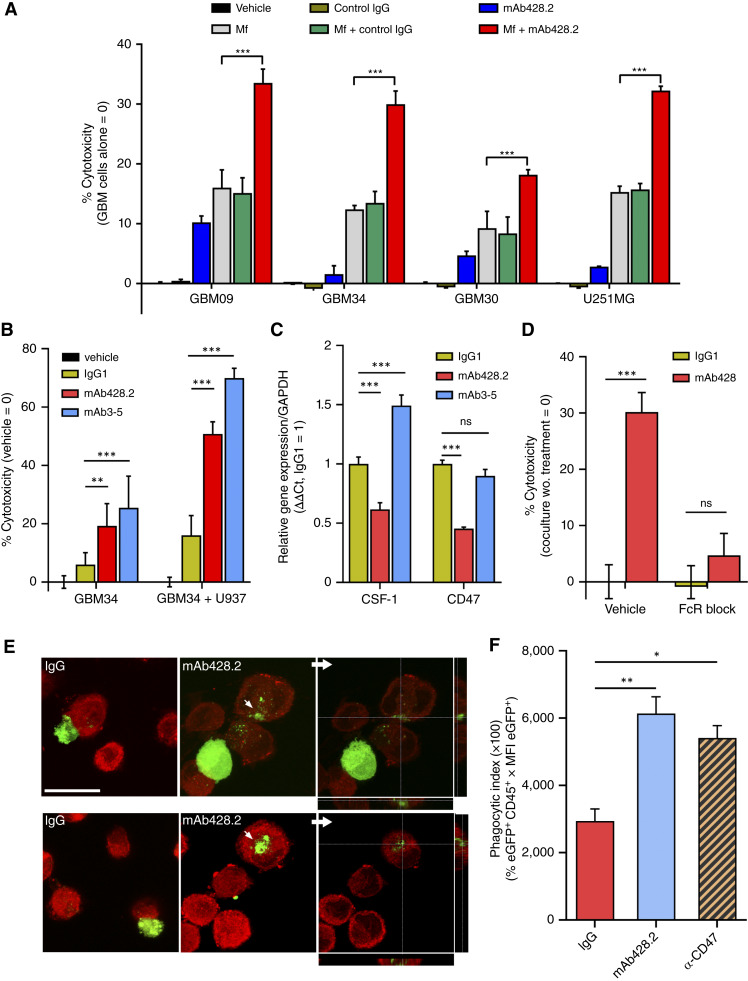
Anti–fibulin-3 induces macrophage attack of GBM cells. **A,** Quantification of cytotoxicity in fLuc-expressing GBM cells co-cultured with U937 macrophages (effector:target = 5:1) in the presence of anti–fibulin-3 mAb428.2, control IgG, or vehicle. Control conditions included GBM cells incubated alone in the presence or absence of the same antibodies. The results were analyzed by one-way ANOVA for each cell line. **B,** Cytotoxicity of GBM cells cocultured with U937 macrophages in the presence of anti–fibulin-3 antibodies mAb428.2 or mAb3-5. **C,** Effect of anti–fibulin-3 antibodies on the expression of immunosuppressive signals by GBM cells. **D,** mAb428.2-induced cytotoxicity of GBM cells (GBM09) cocultured with U937 macrophages that were not pretreated (*vehicle*) or pretreated with excess of IgG-Fc fragment to block immunoglobulin receptors (*FcR block*). The results in (**B–D**) were analyzed by two-way ANOVA. **E,** Representative images of DiI-stained macrophages (red) cocultured with eGFP-expressing GBM9 cells (green) in the presence of mAb428.2 or control IgG. Tumor cell fragments were rarely observed inside macrophages in the control condition, even in direct contact with tumor cells. Confocal sectioning confirmed that eGFP cell fragments were inside the macrophages in presence of mAb428.2; scale bar, 25 μm. **F,** Flow cytometric analysis of phagocytic activity of U937 macrophages cocultured with GBM9:eGFP cells in the presence of control IgG, mAb428.2 (50 μg/mL), or an antibody against the cell surface phagocytosis inhibitor CD47 (10 μg/mL, positive control). The results were analyzed by one-way ANOVA. MFI, mean fluorescence intensity; ns, not significant.

We next tested whether mAb428.2 could modify the behavior of myeloid cells cocultured with GBM cells. Interestingly, fibulin-3 expression increased when GBM cells were exposed to secreted signals from macrophages (Supplementary Fig. S8). However, this did not prevent the tumor cells from being attacked when the anti–fibulin-3 antibody was added to the cocultures. We observed a consistent increase in GBM cell death when several tumor cell lines were cocultured with differentiated U937 macrophages in the presence of mAb428.2, compared with IgG1 or vehicle controls (Fig. 6A). We confirmed that mAb428.2 induced toxicity of different myeloid cells against GBM, including a different macrophage cell line (THP-1), primary macrophages derived from human peripheral blood mononuclear cells or mouse BM, and HMC3 microglial cells (Supplementary Fig. S9).

We also confirmed that antibody-dependent cell cytotoxicity could be induced by a different anti–fibulin-3 antibody (mAb3-5) that did not decrease the expression of immunomodulatory signals in GBM cells (Fig. 6B and C). Furthermore, pretreatment of macrophages with an excess of IgG Fc fragment (100 μg/mL) to saturate immunoglobulin receptors prevented the death of tumor cells in coculture (Fig. 6D), suggesting that the mechanism of death involved macrophages attacking anti–fibulin-3–opsonized tumor cells. We also evaluated mAb428.2 in cocultures of macrophages with the cancer cell line Colo201; these cells do not express fibulin-3, and their death was unaffected by mAb428.2 (Supplementary Fig. S5). Additionally, we tested the cytotoxicity of macrophages against GSCs transfected with fibulin-3 siRNAs. Knockdown of fibulin-3 decreased GSC viability, as we have previously reported (28), but it did not increase the cytotoxic effect of macrophages (Supplementary Fig. S9).

Confocal microscopy of macrophages cocultured with eGFP-expressing GSCs revealed eGFP-positive cell fragments inside the macrophages when mAb428.2 was added to the cultures, compared with control IgG (Fig. 6E), suggesting that the anti–fibulin-3 antibody increases immune attack by phagocytosis/trogocytosis. Accordingly, cocultures were further analyzed by flow cytometry, and a phagocytic index was calculated as the median fluorescence intensity of eGFP detected in CD45^+^ cells. Anti–fibulin-3 mAb428.2 significantly increased the phagocytic index compared with control IgG (Fig. 6F; Supplementary Fig. S10). We further confirmed that mAb428.2 promoted the lysosomal uptake of GBM cargo in macrophages cocultured with GSCs that had been loaded with the pH-sensitive dye pHrodo Green (Supplementary Fig. S10).

Taken together, our results suggest that fibulin-3 targeting decreases immunosuppressive signals in GBM cells, whereas anti–fibulin-3–mediated opsonization allows macrophage attack of the tumor cells, both effects that can be achieved with mAb428.2.

## Discussion

Malignant gliomas contain a complex ECM that combines CNS-specific glycoproteins, secreted by neural cells, with fibrillar proteins produced by the tumor cells and other tumor-associated cells that contribute to a mesenchymal phenotype (36, 37). The result is a unique tissue scaffold that is different from the mucinous ECM of normal brain parenchyma and the fibrillar ECM scaffold of other solid tumors. Therefore, it is not surprising that ECM proteins produced “*de novo*” by GBM cells have a significant impact on processes such as tumor cell invasion, neovascularization, astrocytic reactivity, and regulation of immune cell responses in the neural microenvironment. Anti-ECM approaches have shown efficacy against GBM, either by direct effect on the tumor cells (26, 38, 39) or as adjuvant treatment to improve the penetration of biological agents, including immune cells (20, 40). Antibodies targeting ECM proteins have successfully reached clinical trials for GBM, such as *tenatumomab* against tenascin-C (41) and *L19* against a variant of fibronectin (42), although they depend on conjugated payloads for antitumor effect. AntipHrodo Green fibulin-3 mAb428.2 aligns with these approaches, with the added advantage of directly blocking fibulin-3 signaling (26) to elicit deleterious effects on tumor cells.

In the present study, we have shown that fibulin-3, which contributes to the ECM meshwork around GBM cells, has a novel role limiting the ability of myeloid cells to act against the tumor. This effect is exerted, in part, by an autocrine loop in which fibulin-3 activates NF-κB–mediated transcription in GBM cells to increase their expression of signals that modify TAM behavior, such as CSF-1, TGF-β1, and the phagocytosis inhibitor CD47. This is the first observation of a “small fibulin” member (i.e., fibulin-3, -4, and -5) as an immunomodulatory factor. The ability of fibulin-3 to indirectly modify TAM behavior is particularly interesting because of the spatial localization of this protein in GBM tumors. Fibulin-3 is enriched in perinecrotic areas of the tumor core, accumulating around blood vessels that are the route of entry of BM-derived macrophages (28, 34). Tumor areas rich in fibulin-3 would therefore be primed to induce macrophage immunosuppression upon infiltration, contributing to the rapid hijacking of this immune population. This effect likely extends to local microglia within the tumor, as we observed similar reactivation changes in the macrophage (CD45^hi^ CD11b^+^) and microglia (CD45^lo^ CD11b^+^) populations after fibulin-3 knockdown *in vivo*. Furthermore, anti–fibulin-3–targeting induced both macrophages and microglia to attack tumor cells, suggesting a common immunomodulatory effect of fibulin-3 on different myeloid populations in GBM.

The regulation of multiple myeloid-suppressive signals by fibulin-3 is of particular interest because it suggests that targeting this ECM protein can disrupt redundant mechanisms that promote TAM immunosuppression. This may be worth comparing against CSF-1 receptor inhibition, which prevents the polarizing effect of this cytokine on TAMs (43) but results in upregulation of insulin-like growth factor-1 (IGF-1) signaling that promotes tumor relapse (44). Downregulation of CSF-1 in GBM cells by fibulin-3 inhibition did not lead to increased IGF-1 in the tumor (Supplementary Fig. S7), possibly preventing this tumor-salvage pathway. Whether this reveals different effects of inhibiting CSF-1R compared with decreasing CSF-1 in the tumor, or whether the lack of IGF-1 upregulation reflects a limitation of our athymic model due to lack of T-cell signaling, remains to be investigated. Another important effect of fibulin-3 is the regulation of the checkpoint CD47 that inhibits macrophage phagocytosis (45). CD47 expression correlates with poor prognosis in solid tumors, including glioma (46, 47), and antibody-mediated targeting of this receptor increases tumor attack by myeloid cells (48). Targeting fibulin-3 to downregulate CD47 in the GSC population could prove a useful complementary approach to promote immune reactivation against the tumor. It is possible, however, that this effect could be limited by other innate checkpoint molecules, such as CD24 and B2M, that were not affected by fibulin-3 knockdown in GSCs (Supplementary Fig. S4).

It is worth noting that inhibition of fibulin-3 altered the expression of several immunomodulatory molecules, and therefore the effects of this protein should not be interpreted by assessing individual cytokine changes. For example, inhibition of fibulin-3 in GSCs reduced the expression of CSF-1, which promotes macrophage migration, but also decreased other signals that prevent myeloid infiltration and activation (e.g., MIF, Fig. 3C). Similarly, fibulin-3 inhibition decreased IL-1β in the tumor cells (Fig. 2C) but IL-1β in the host (mouse) was unchanged or increased (Fig. 1C; Supplementary Fig. S7), indicating additional cell sources for this proinflammatory cytokine in the tumor (49). Furthermore fibulin-3 inhibition increased tumor cell death [Supplementary Fig. S9 and previously reported (28)], likely releasing damage-associated molecules. Therefore, the increased presence and antitumor activation of TAMs following fibulin-3 knockdown or antibody blocking should be considered the net result from multiple deleterious and proinflammatory effects of targeting this protein.

We recognize several limitations in this study, which will require further investigation to better understand the immunomodulatory role of fibulin-3 in GBM. Most importantly, this study has not addressed the possible direct effects of fibulin-3 on myeloid or other immune cells. Future studies should investigate these effects *in vivo*, including comprehensive transcriptomic identification and characterization of immune cell populations affected by fibulin-3. In addition, our analyses were performed at defined experimental time points with limited immunologic markers (IBA1 and CD206), which may not have captured the temporal changes in immune composition and phenotype during tumor growth. Further studies should also confirm *in vivo* our observations *in vitro*, including the antitumor polarization of TAMs, their cytokine profile, and their increased cytotoxic/phagocytic activity elicited by targeting fibulin-3 in GBM cells.

In conclusion, the work presented in this article shows that fibulin-3, an ECM protein highly upregulated in malignant gliomas, is an important immunomodulatory component in these tumors. Fibulins, chondroitin sulfate proteoglycans, hyaluronic acid, tenascins, fibronectin, osteopontin, and collagens have been recognized as modulators of immune responses (16, 50), either by direct effect in immune cells, activation of immunosuppressive mechanisms in tumor cells, or changing the mechanical properties of the tissue scaffold (51). We propose that fibulin-3, as well as other pericellular ECM proteins, can be inhibited to reduce immunosuppression in GBM and should be explored as adjuvant targets for successful immunotherapy of these aggressive brain tumors.

## Supplementary Material

Supplementary Figure Legends Supplementary Figure Legends 1-10 and Supplementary Tables 1-2.

Supplementary Figure S1 Figure S1. Validation of GBM stem cells and equivalence of humanized mAb428.2 to original mAb428 antibody.

Supplementary Figure S2 Figure S2. Analysis of TAMs in intracranial tumors by quantitative immunohistochemistry.

Supplementary Figure S3 Figure S3. Correlation of fibulin-3 expression with immunosuppressive signals in GBM.

Supplementary Figure S4 Figure S4. Fibulin-3 knockdown does not affect the expression of some immune checkpoints.

Supplementary Figure S5 Figure S5. Characterization of fibulin-3 effects and anti-fibulin-3 targeting in fibulin-3-null cells.

Supplementary Figure S6 Figure S6. Fibulin-3 induces p65/RelA binding to the CSF1 promoter.

Supplementary Figure S7 Figure S7. Anti-fibulin-3 treatment decreases immunosuppression in GBM.

Supplementary Figure S8 Figure S8. Fibulin-3 is upregulated by macrophage signals.

Supplementary Figure S9 Supplementary Figure S9. Anti-fibulin-3 triggers myeloid cell attack against syngeneic GBM cells.

Supplementary Figure S10 Figure S10. Anti-fibulin-3 promotes in vitro phagocytosis of GBM cells by macrophages.

Supplementary Table S-I Table S-I. Antibodies used for Western blotting, flow cytometry, or immunohistochemistry.

Supplementary Table S-II Table S-II. Sequences of oligonucleotides and primers used for q-RTPCR.
