# Supplementary material for: Inhibition of Extracellular Matrix Protein Fibulin-3 Reduces Immunosuppressive Signaling and Increases Macrophage Activation in Glioblastoma
Source: Cancer Res Commun. 2025 Sep 11;5(9):1599–610. doi: 10.1158/2767-9764.CRC-25-0083 (PMC12423750; doi:10.1158/2767-9764.CRC-25-0083)
Supplement: Supplementary Figure Legends — 1-10 and Supplementary Tables 1-2. [file crc-25-0083_supplementary_figure_legends_suppsfl_1-10stl_1-2.pdf]

Inhibition of extracellular matrix protein fibulin-3 reduces immunosuppressive signaling and increases macrophage activation in glioblastoma

Somanath Kundu, Soham Mitra, Arivazhagan Roshini, John A. Longo, Sharon L. Longo, Abigail Venskus, Harish Babu, Mariano S. Viapiano

Supplementary Materials:

Supplementary Tables S-I and S-II

Supplementary Figures S1 to S10

Supplementary Table S-I:

Antibodies used for Western blotting, flow cytometry, or immunohistochemistry

Supplementary Table S-II:

Sequences of oligonucleotides and primers used for q-RT-PCR

Suppl. Figure S1: *Validation of GBM stem cells and equivalence of humanized mAb428.2 to original mAb428 antibody.* **A)** A collection of 44 GBM stem cells (GSCs) prepared in the Viapiano laboratory were profiled by RNAseq and the resulting gene expression counts were normalized using the Trimmed Mean of M-values (TMM) method. Expression of the immunosuppressive signals CSF-1 and CD47 in these cells was plotted against fibulin-3. The GSCs chosen for this study are representative of a linear range of expression for fibulin-3 and its correlated genes. **B)** Using the original V_H and V_L sequences of mouse mAb428.2 (Nandhu et al., Clin. Cancer Res. 2018), five humanized V_H and V_L variants were generated and cloned in a human IgG1 backbone

(Absolute Antibody Ltd.), generating a total of 25 humanized mAb428.2 variants (*v1-v25*). All the variants were expressed in HEK293 cells, purified from culture medium to homogeneity, and tested against human fibulin-3 by indirect ELISA. The graph represents production yield of humanized mAb428.2 variants (expressed as fold over the production of mouse chimeric mAb428.2 in HEK293 cells) and their binding affinity for fibulin-3. The red open dot represents the mouse monoclonal antibody. **C)** Percent of each humanized variant that remains as stable monomeric IgG in phosphate-buffered saline solution, compared against the mouse antibody source (*chimeric*). The inset images show non-reducing and reducing SDS-PAGE of purified mouse chimeric mA428.2 antibody (including a minor unstable product, arrow) and the humanized mAb428.2.v10 chosen for this study. **D)** Representative Western blots of purified fibulin-3 (100 ng) detected with each humanized variant (tested at a concentration of 1 µg/mL). **E)** Representative ELISAs of the three humanized variants chosen for production (v5, v7, v10) because of their high production yield and high affinity against fibulin-3 compared to the original mouse antibody. The humanized variant hmAb428.2.v10 was used in the present study.

Suppl. Figure S2: *Analysis of TAMs in intracranial tumors by quantitative immunohistochemistry.* **A)** Comparison of intracranial tumor models with fibulin-3 knockdown versus their controls. **B)** Comparison of intracranial tumor models with fibulin-3 overexpression versus their controls. **C)** Comparison of an intracranial tumor model treated with anti-fibulin-3 mAb428.2 versus control IgG. For each tumor model, the results shown are as follows (left to right): 1) Total TAM density ($IBA1^+$ area in the tumor); 2) IBA/CD206 co-expression in TAMs; 3) number of cells per mm^2 (cell density control); and 4) area analyzed for each tumor section (tumor area control). Results for each parameter were analyzed by Student's t-test with Welch's correction.

Suppl. Figure S3: *Correlation of fibulin-3 expression with immunosuppressive signals in GBM.* **A)** Correlation of fibulin-3 expression with immunosuppressive signals (IL-10, TGFβ2, TGFβ3)

and checkpoint molecules (CD80, CD86, CD24, B2M), extracted from the TCGA GBM dataset. **B)** Correlation of fibulin-3 expression with an immunosuppressive signature (CSF-1, TGFβ1, IL-10, CD47, CD274, CD86) observed in the CGGA GBM dataset. Data from the TCGA and CGGA datasets were recovered from the aggregator website Gliovis.

Suppl. Figure S4: *Fibulin-3 knockdown does not affect the expression of some immune checkpoints*. Two GBM stem cell lines (GBM09 and GBM34) were stably transfected with control or fibulin-3 shRNAs and subjected to RNAseq analysis, as shown in **Figure 2C**. Gene expression counts (normalized by TMM method and expressed as fold change to control) were compared for the innate immune checkpoints CD24 and B2M. Results did not show significant differences between control and fibulin-3-knockdown conditions (data analyzed by two-way ANOVA).

Suppl. Figure S5: *Characterization of fibulin-3 effects and anti-fibulin-3 targeting in fibulin-3-null cells*. **A)** The colorectal carcinoma cell line Colo201, which lacks endogenous fibulin-3 expression, was transfected with control or fibulin-3 cDNA and processed for qRT-PCR. Results show that forced overexpression of fibulin-3 in these cells increases their expression of immunosuppressive signals. **B)** Colo201 cells were cultured alone or with U937 macrophages in presence of anti-fibulin-3 antibody, mAb428.2, or control IgG. Results show a high baseline cytotoxicity caused by the macrophages (probably due to low expression of CD47 in Colo201), which was not increased by mAb428.2. Results in (**A**) and (**B**) were analyzed by two-way ANOVA.

Suppl. Figure S6: *Fibulin-3 induces p65/RelA binding to the CSF1 promoter.* GSCs (GBM09 line) were treated with vehicle, fibulin-3 (500 ng/mL), or the strong NF-κB activator TNF-α (50 ng/mL) for 2 hours. Cells were subsequently processed for chromatin immunoprecipitation (ChIP) using a ChIP-validated antibody against the canonical NF-κB transcription factor p65/RelA

(Sigma-Aldrich cat #17-10060), following the manufacturer's instructions. qPCR primers (listed in Suppl. Table II) were designed to flank the sequence GGAAAGTCCC (location -377 to -368) that is the known NF-κB binding sequence in the promoter of *CSF1* gene (Yamada et al., Blood (1991) 78:1988). Primers against the *NFKBIA* gene promoter (IκBα, positive control target) were provided in the ChIP kit. For each cell treatment, the qPCR signal was quantified in the input material (total fragmented chromatin) and in the p65-precipitated chromatin fragments (ChIP-qPCR). Quantification of *CSF1* and *NFKBIA* promoter DNA precipitated by p65 was normalized to the input material and expressed as fold change from untreated cells. **A)** The results indicate significantly increased binding of p65 to *CSF1* and *NFKBIA* promoters in GSCs treated with fibulin-3 (data analyzed by two-way ANOVA), confirming that fibulin-3 activates NF-κB-mediated transcription. **B)** Agarose gel showing the detection of input and immunoprecipitated DNA after PCR amplification.

Suppl. Figure S7: *Anti-fibulin-3 treatment decreases immunosuppression in GBM.* Intracranial tumors (GBM09 model) treated locally with mAb428.2 (shown in **Figure 5**) were freshly resected and processed for qRT-PCR utilizing mouse-specific primers. Results show the comparative mRNA expression of several genes associated with TAM immunosuppression (CD206, ARG1, CD163), pro-inflammatory TAM phenotype (TNF-α, IL-1β), and pro-tumoral phenotype observed in macrophages associated with rebound GBM (IGF-1, Quail et al., Science (2016) 352:aad3018). Results were compared by multiple paired t-tests with correction for multiple comparisons.

Suppl. Figure S8: *Fibulin-3 is upregulated by macrophage signals.* GSCs were cultured for 24 hours in basal culture medium alone or supplemented with serum-free conditioned medium from bone marrow-derived macrophages. Cells were subsequently processed for qRT-PCR. Results

show a significant increase of fibulin-3 mRNA expression in all the GSC lines exposed to macrophage conditioned medium. Results were analyzed by two-way ANOVA.

Suppl. Figure S9: *Anti-fibulin-3 triggers myeloid cell attack against syngeneic GBM cells*. **A)** fLuc-expressing GBM cells (GBM09, GBM34, and GL261) were co-cultured with a macrophage cell line (THP-1) or primary macrophages, as indicated in the figure panels, in presence of anti-fibulin-3 mAb428.2 or its control IgG. The results show the extent of tumor cell death (quantified by decrease of fLuc signal) after 48 h of co-culture. Data were normalized to fLuc signal from cultures of GBM cells alone, treated with the same antibodies. Results were analyzed by Student's t-test for each GBM cell line. *PBMC:* macrophages derived from peripheral blood mononuclear cells; *BMDM:* bone marrow-derived macrophages. **B)** fLuc-expressing GBM cells (GBM09, GBM34) were co-cultured with HMC3 microglia in presence of anti-fibulin-3 mAb428.2, control IgG1, or vehicle. The results show tumor cell cytotoxicity after 48 h, normalized to a culture of GBM cells in absence of other cells or treatments. Results were analyzed by one-way ANOVA for each GBM cell line. **C)** fLuc-expressing GBM34 cells were transiently transfected with control or fibulin-3 siRNAs. The following day they were co-cultured with U937 macrophages for an additional 48h. Viability of fibulin-3 knockdown cells was compared against control cells, either alone or in coculture. Results show a cytotoxic effect of fibulin-3 knockdown in GSCs, which was not increased significantly by macrophages (results analyzed by two-way ANOVA). Note that the baseline viability is calculated as 100% for the control cells in both cultures shown in this figure panel; this is to compensate for the different growth rates of GBM cells alone and co-cultured with macrophages –in absence of other treatments.

Suppl. Figure S10: *Anti-fibulin-3 promotes in vitro phagocytosis of GBM cells by macrophages.* **A)** Representative flow cytometry gating of eGFP-expressing GSCs phagocytosed by macrophages in presence of control IgG, anti-fibulin-3 mAb428.2, or positive control anti-CD47

antibody (quantification shown in Figure 6F). **B-D**) Confirmation of phagocytosis using pHrodo-loaded GSCs co-cultured with macrophages. Non-fluorescent GSCs were incubated with pHrodo-green following the manufacturer's instructions, washed, and co-cultured with macrophages for 120 min in presence of control IgG or anti-fibulin-3 mAb428.2 (50 µg/mL). Cells were assessed by flow cytometry and a phagocytic index was calculated as indicated in Figure 6F. The panels show representative flow cytometry assays (**B**), quantitative analysis (**C,** $p<0.05$ by Student's t-test), and visualization of lysosomal uptake of GBM cargo in the macrophages (**D**, bar = 20 µm). A proportion of GBM cells were also stained with pHrodo during the co-culture.
