# Supplementary figures and images for: Inhibition of Extracellular Matrix Protein Fibulin-3 Reduces Immunosuppressive Signaling and Increases Macrophage Activation in Glioblastoma
Source: Cancer Res Commun. 2025 Sep 11;5(9):1599–610. doi: 10.1158/2767-9764.CRC-25-0083 (PMC12423750; doi:10.1158/2767-9764.CRC-25-0083)

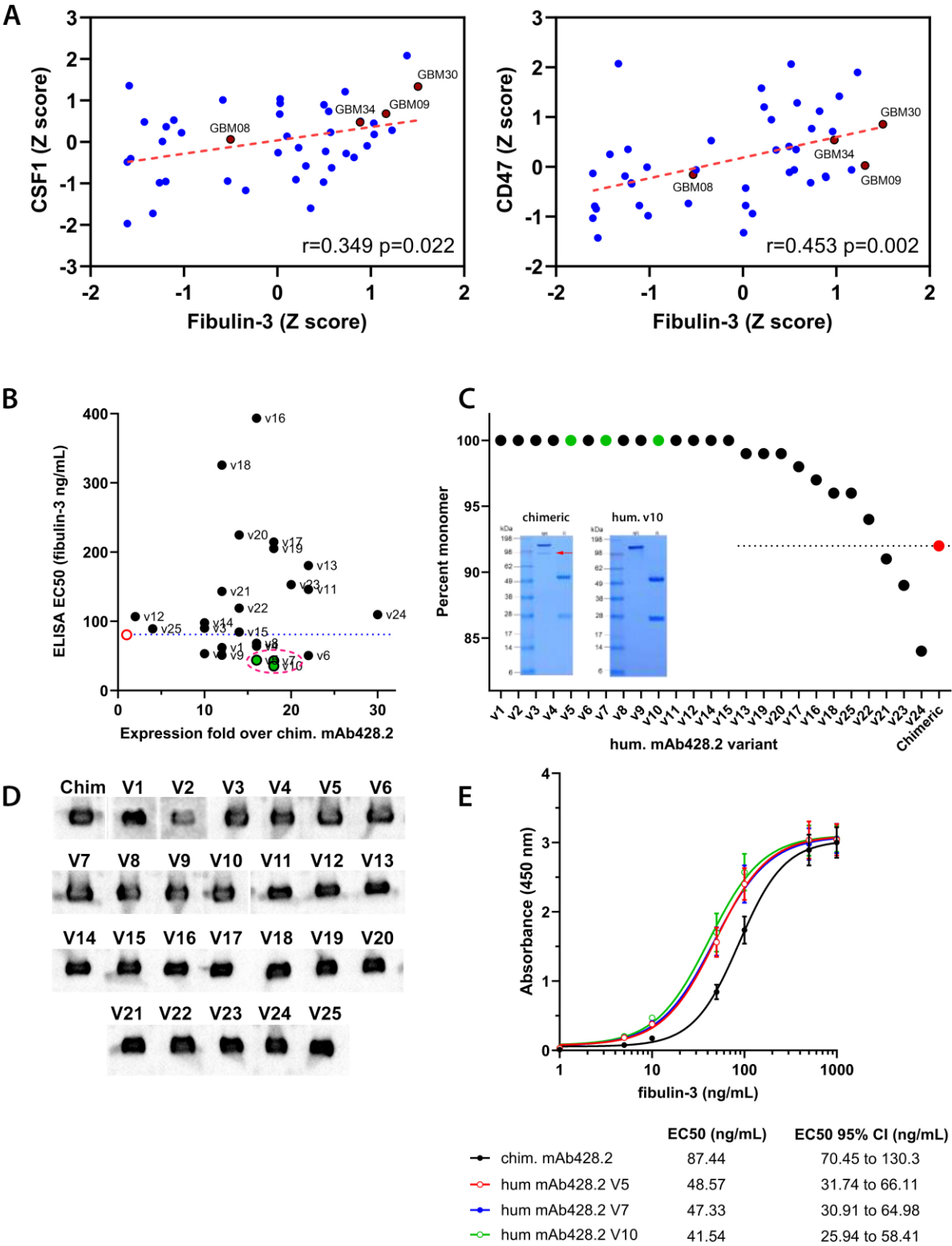

Supplement: Supplementary Figure S1 — Figure S1. Validation of GBM stem cells and equivalence of humanized mAb428.2 to original mAb428 antibody. [file crc-25-0083_supplementary_figure_s1_suppsf1.pdf]

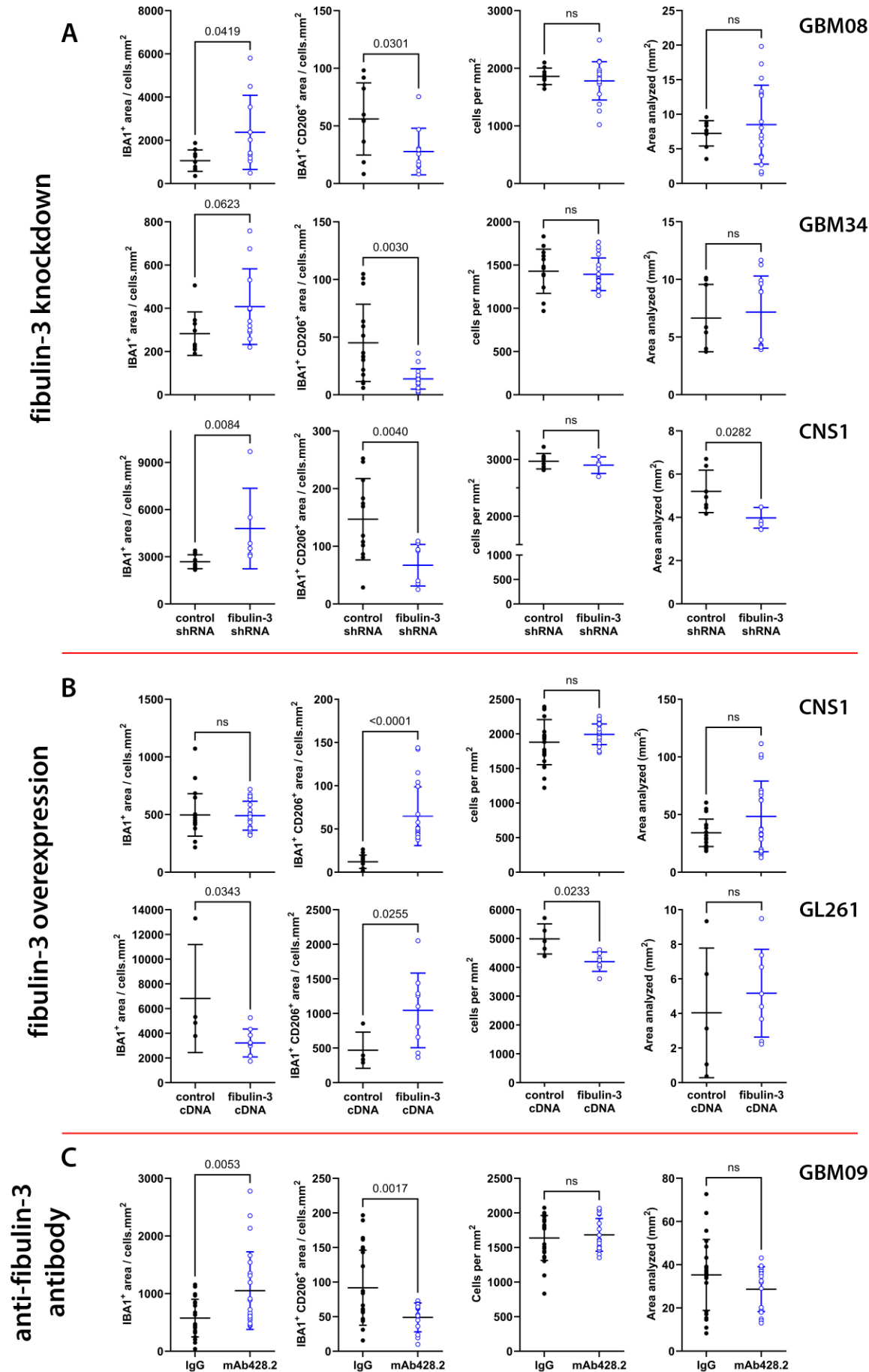

Supplement: Supplementary Figure S2 — Figure S2. Analysis of TAMs in intracranial tumors by quantitative immunohistochemistry. [file crc-25-0083_supplementary_figure_s2_suppsf2.pdf]

## A) TCGA

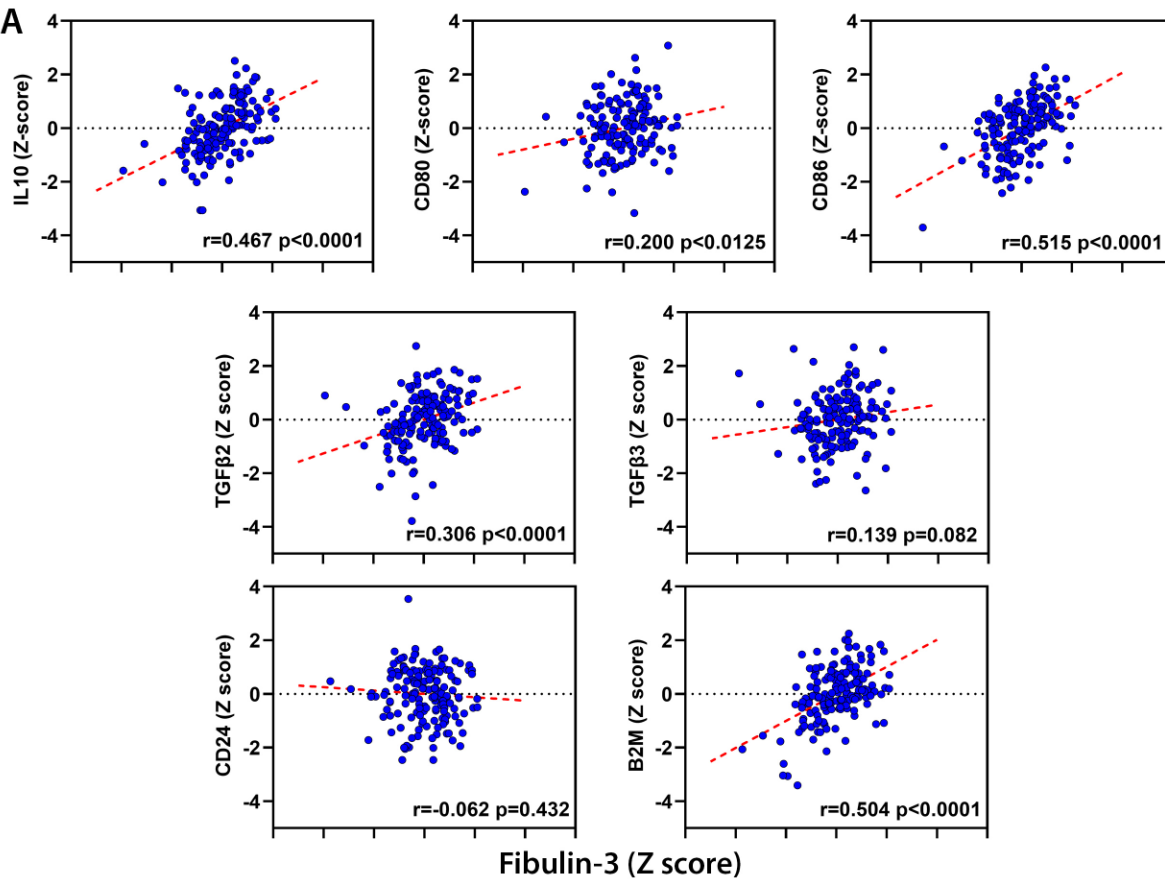

## B) CGGA

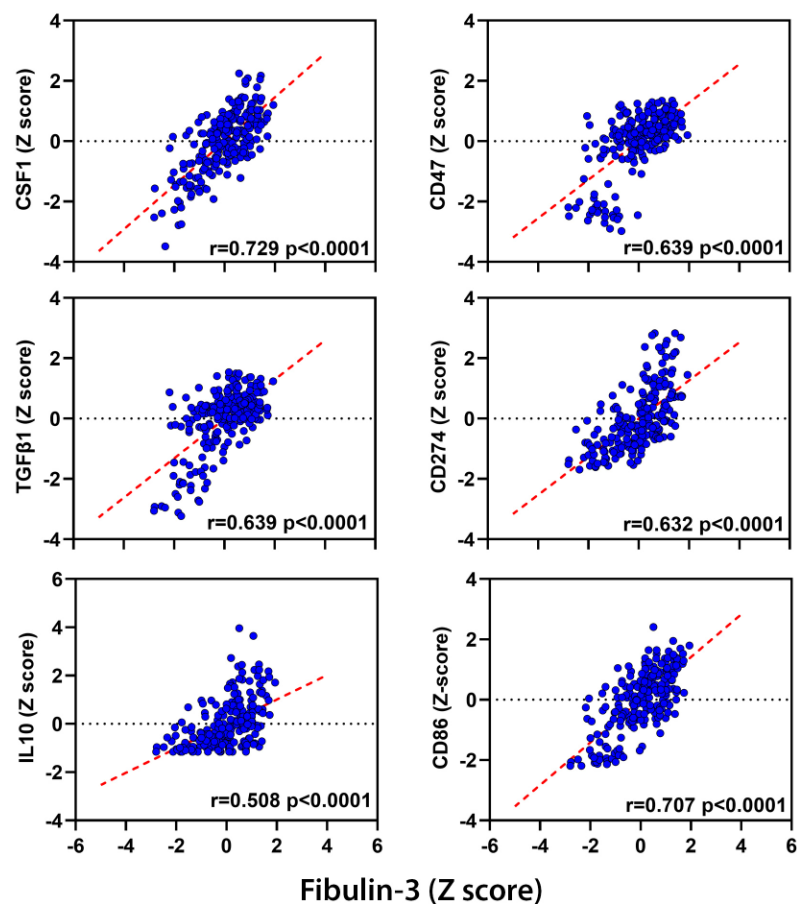

Supplement: Supplementary Figure S3 — Figure S3. Correlation of fibulin-3 expression with immunosuppressive signals in GBM. [file crc-25-0083_supplementary_figure_s3_suppsf3.pdf]

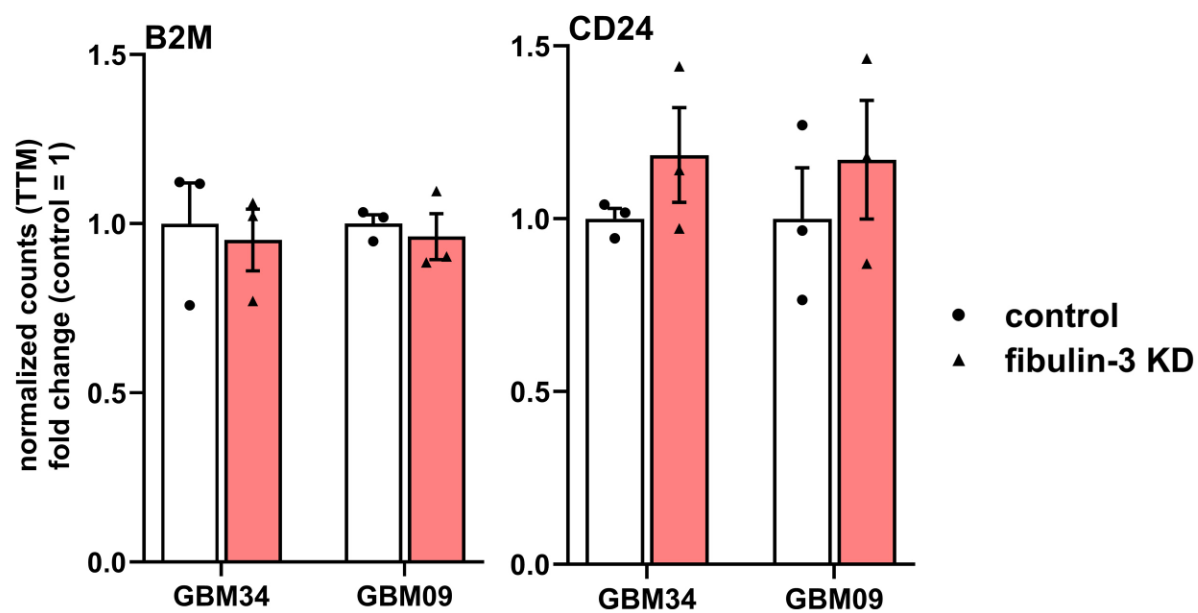

Supplement: Supplementary Figure S4 — Figure S4. Fibulin-3 knockdown does not affect the expression of some immune checkpoints. [file crc-25-0083_supplementary_figure_s4_suppsf4.pdf]

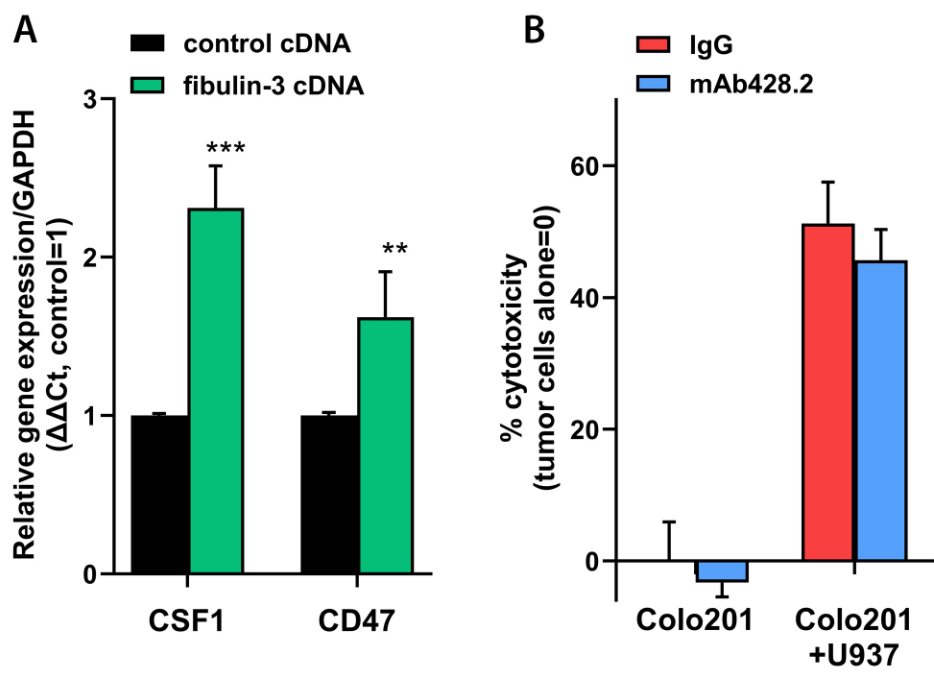

Supplement: Supplementary Figure S5 — Figure S5. Characterization of fibulin-3 effects and anti-fibulin-3 targeting in fibulin-3-null cells. [file crc-25-0083_supplementary_figure_s5_suppsf5.pdf]

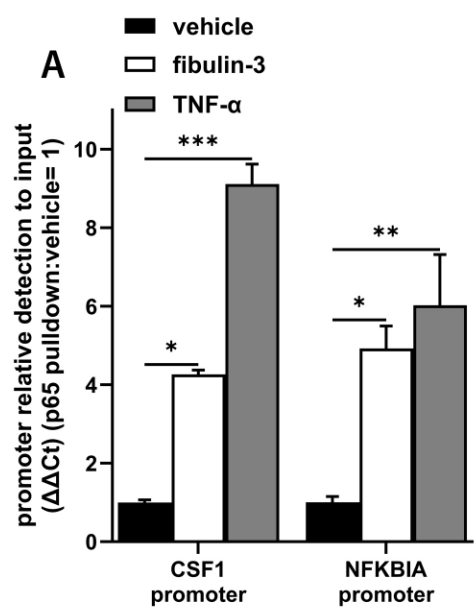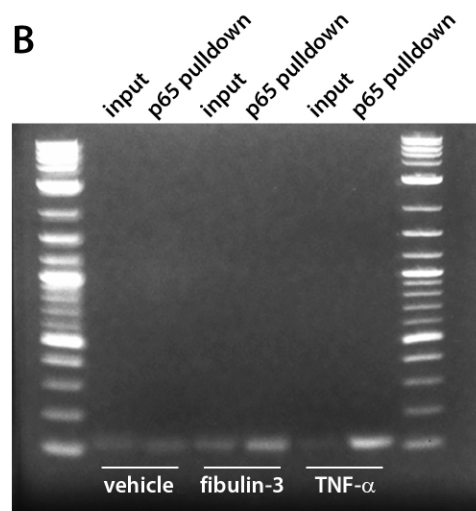

Supplement: Supplementary Figure S6 — Figure S6. Fibulin-3 induces p65/RelA binding to the CSF1 promoter. [file crc-25-0083_supplementary_figure_s6_suppsf6.pdf]

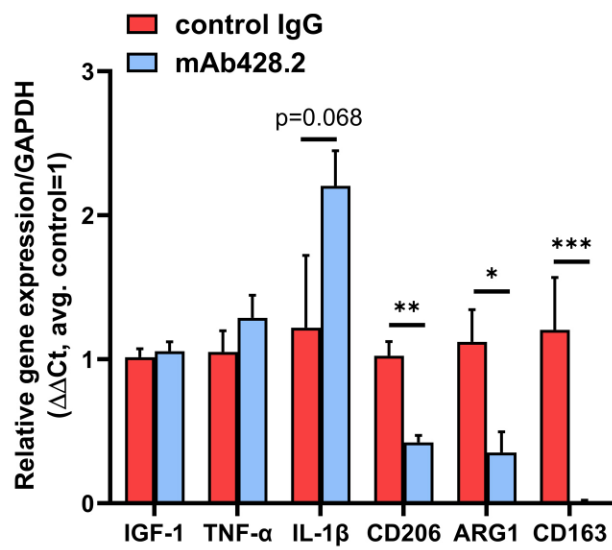

Supplement: Supplementary Figure S7 — Figure S7. Anti-fibulin-3 treatment decreases immunosuppression in GBM. [file crc-25-0083_supplementary_figure_s7_suppsf7.pdf]

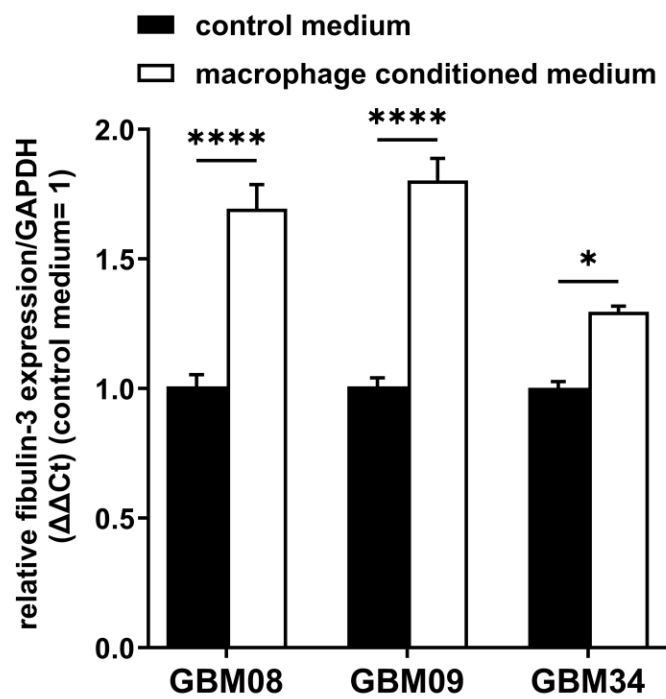

Supplement: Supplementary Figure S8 — Figure S8. Fibulin-3 is upregulated by macrophage signals. [file crc-25-0083_supplementary_figure_s8_suppsf8.pdf]

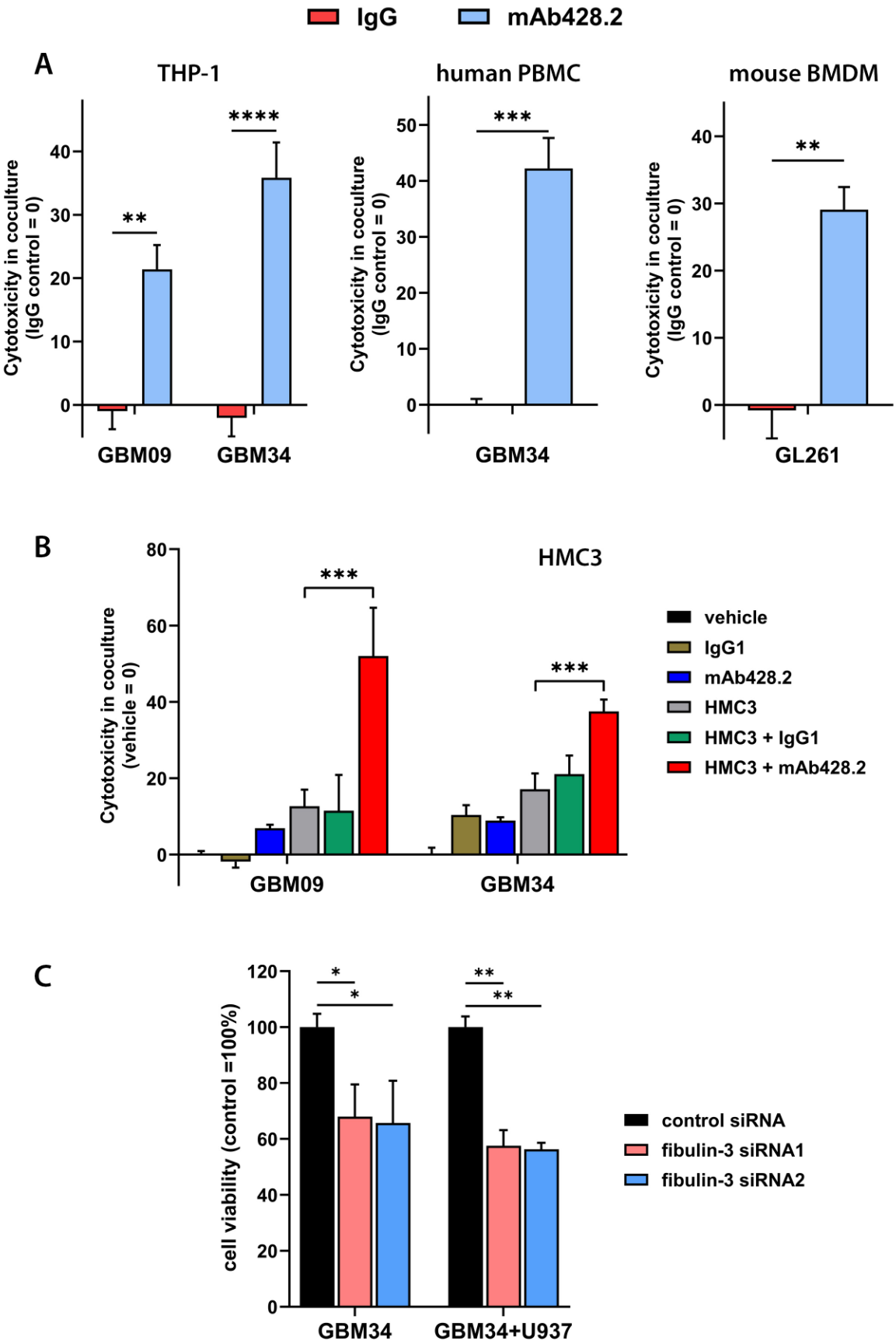

Supplement: Supplementary Figure S9 — Anti-fibulin-3 triggers myeloid cell attack against syngeneic GBM cells. [file crc-25-0083_supplementary_figure_s9_suppsf9.pdf]

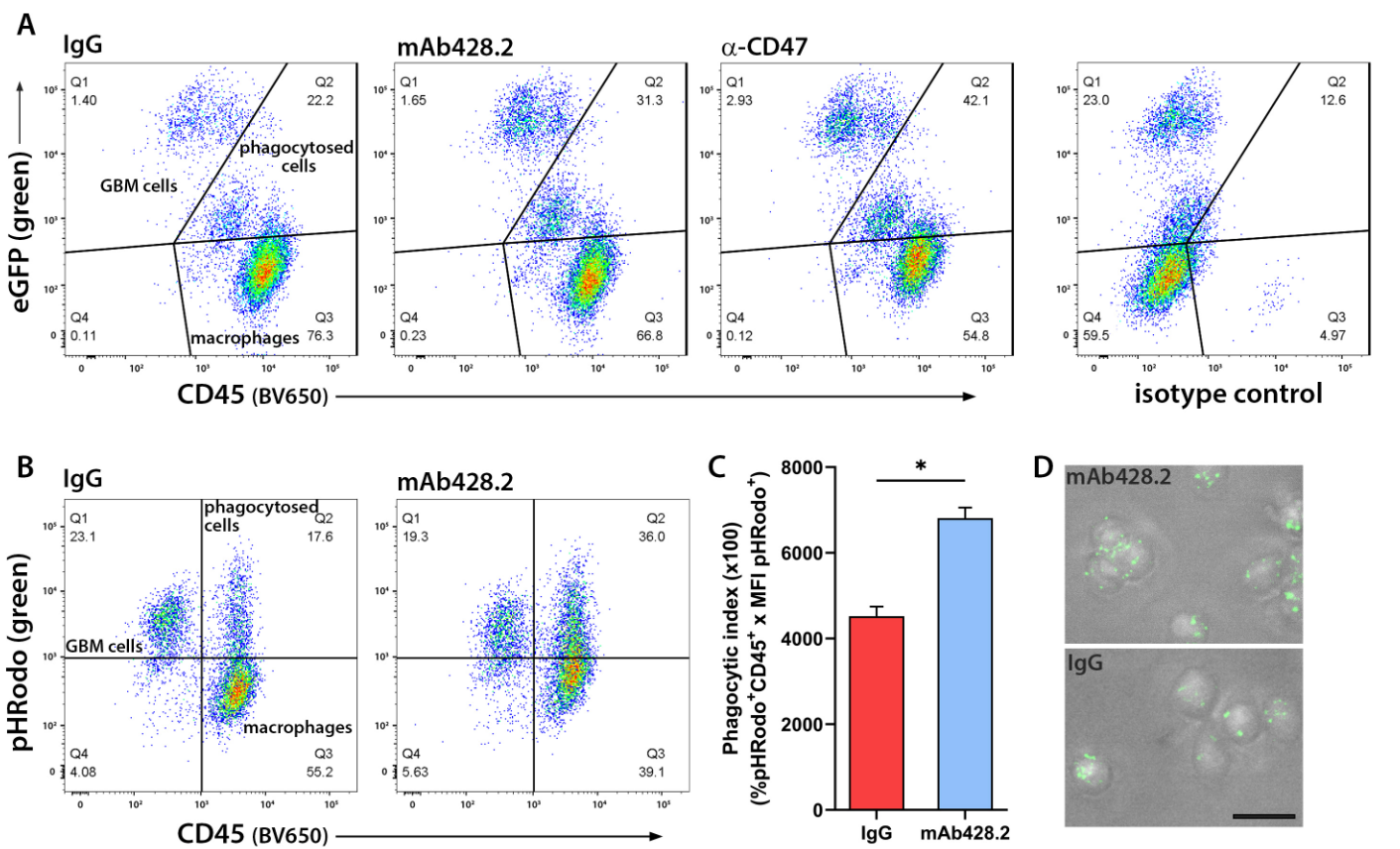

Supplement: Supplementary Figure S10 — Figure S10. Anti-fibulin-3 promotes in vitro phagocytosis of GBM cells by macrophages. [file crc-25-0083_supplementary_figure_s10_suppsf10.pdf]
