# Supplementary material for: Inhibition of Extracellular Matrix Protein Fibulin-3 Reduces Immunosuppressive Signaling and Increases Macrophage Activation in Glioblastoma
Source: Cancer Res Commun. 2025 Sep 11;5(9):1599–610. doi: 10.1158/2767-9764.CRC-25-0083 (PMC12423750; doi:10.1158/2767-9764.CRC-25-0083)
Supplement: Supplementary Table S-I — Table S-I. Antibodies used for Western blotting, flow cytometry, or immunohistochemistry. [file crc-25-0083_supplementary_table_s-i_suppst1.pdf]

Kundu et al.

Supplementary table I:

Antibodies used for Western blotting, flow cytometry, or immunohistochemisty

Antibody	Host Species	Source	Catalog #	Usage	RRID
CD11b conj. PE	Human	Miltenyi Biotec	#130-113-806	Flow cytometry	AB_2751172
CD206 conj. BV421	Rat	BioLegend	#141717	Flow cytometry	AB_2562232
CD45 conj. APC	Rat	BioLegend	#103112	Flow cytometry	AB_312977
CD45 conj. BV650	Mouse	BioLegend	#304043	Flow cytometry	AB_2562498
Isotype control APC	Rat	Bio Legend	#400611	Flow cytometry	AB_326555
Isotype control BV421	Rat	Bio Legend	#400535	Flow cytometry	AB_10933427
Isotype control BV650	Mouse	BioLegend	#400163	Flow cytometry	AB_11126986
isotype control PE	Human	Miltenyi Biotec	#130-113-450	Flow cytometry	AB_2733892
CD16/CD32 (Fc block)	Rat	BioLegend	#101302	Flow cytometry	AB_312801
CD206	Goat	R&D Systems	#AF2535	IHC	AB_2063012
IBA1	Rabbit	Wako	#019-1974	IHC	AB_839504
CD47	Rabbit	Cell Signaling Technology	#63000	Western blotting	AB_2799637
CD47	Mouse	BioXcell	BE0019-1	Flow cytometry	AB_1107655
CSF-1	Mouse	Santa Cruz Biotechnology	sc-365779	Western blotting	AB_10846852
Fibulin-3	Mouse	Santa Cruz Biotechnology	#sc-33722 (mAb3-5)	Western blotting	AB_2277777
phospho NF-kB P65	Rabbit	Cell Signaling Technology	#3033	Western blotting	AB_331284
total NF-kB P65	Rabbit	Cell Signaling Technology	#8242	Western blotting	AB_10859369
Vinculin	Mouse	R&D Systems	#MAB6896	Western blotting	AB_10992930
