# Supplementary material for: Inhibition of Extracellular Matrix Protein Fibulin-3 Reduces Immunosuppressive Signaling and Increases Macrophage Activation in Glioblastoma
Source: Cancer Res Commun. 2025 Sep 11;5(9):1599–610. doi: 10.1158/2767-9764.CRC-25-0083 (PMC12423750; doi:10.1158/2767-9764.CRC-25-0083)
Supplement: Supplementary Table S-II — Table S-II. Sequences of oligonucleotides and primers used for q-RTPCR. [file crc-25-0083_supplementary_table_s-ii_suppst2.pdf]

Kundu et al.

Supplementary table II:

Sequences of oligonucleotides and primers used for q-RTPCR

Gene	Oligo / Primer	Human-specific or multispecies sequence
Fibulin-3/EFEMP1	siRNA1	5'- CACGCAATGCCACTGACGGATA
Fibulin-3/EFEMP1	siRNA2	5'- CACAACGTGTGCCAAGACATA
p65/RelA	siRNA1	5'- AAGATCAATGGCTACACAGGA
p65/RelA	siRNA2	5'- CCGGATTGAGGAGAAACGTAA
CD47	Forward	5'- ATGCATGGCCCTCTTCTGA
CD47	Reverse	5'- TTTGAATGCATTAAGGGGTTCCT
CD80	Forward	5'- GGCCCGAGTACAAGAACCG
CD80	Reverse	5'- TCGTATGTGCCCTCGTCAGAT
CSF-1	Forward	5'- CCAGCAACTGGAGAGGTGTC
CSF-1	Reverse	5'- GCAGCTGCAGGAACTCTCTT
Fibulin-3/EFEMP1	Forward	5'- TTTTGCTGTGCTGTGCAAGG
Fibulin-3/EFEMP1	Reverse	5'- CAGTGCATTGCGTGTACGTG
MIF	Forward	5'- CGGACAGGGTCTACATCAACTA
MIF	Reverse	5'- TCTTAGGCGAAGGTGGAGTT
p65/RelA	Forward	5’- ACTGCCGAGCTCAAGATCTG
p65/RelA	Reverse	5’- TCCCGTGAAATACACCTCAA
CD274	Forward	5'- TGGCATTTGCTGAACGCATTT
CD274	Reverse	5'- TGCAGCCAGGTCTAATTGTTTT
TGFβ1	Forward	5'- CAATTCCTGGCGATACCTCAG
TGFβ1	Reverse	5'- GCACAACTCCGGTGACATCAA
GAPDH	Forward	5'- TTGCCCTCAACGACCACTTT
GAPDH	Reverse	5'- TGGTCCAGGGGTCTTACTCC
18S RNA	Forward	5'- AACTTTCGATGGTAGTCGCCG
18S RNA	Reverse	5'- CCTTGGATGTGGTAGCCGTTT
CSF-1 promoter	Forward	5’- AAAGGATTTCCCTCCCTTCC
CSF-1 promoter	Reverse	5’- CAGTGCTAGCGCTCTATGAT
IκBα promoter	Forward	5’- GACGACCCCAATTCAAATCG
IκBα promoter	Reverse	5’- TCAGGCTCGGGGAATTTCC
**Gene**	**Primer**	**Mouse-specific Sequence**
ARG1	Forward	5'- TCACCTGAGCTTTGATGTCG

ARG1	Reverse	5'- CTGAAAGGAGCCCTGTCTTG
CD163	Forward	5’- TCTCCACACGTCCAGAACAG
CD163	Reverse	5’- CCTCGTCACCTTGGAAACAG
CD206	Forward	5'- AGTGGCAGGTGGCTTATG
CD206	Reverse	5'- GGTTCAGGAGTTGTTGTGG
CD47	Forward	5’- AAATGGATAAGCGCGATGCC
CD47	Reverse	5’- GGCTGATCCTTGGTCAGTGT
CSF-1	Forward	5’- GTGTCAGAACACTGTAGCCAC
CSF-1	Reverse	5’- TCAAAGGCAATCTGGCATGAAG
IGF1	Forward	5’- GCAACACTCATCCACAATGC
IGF1	Reverse	5’- AGCTGGACCAGAGACCCTTT
IL-1β	Forward	5'- GCTTCAGGCAGGCAGTATC
IL-1β	Reverse	5'- AGGATGGGCTCTTCTTCAAAG
TNF-α	Forward	5'- CCACCACGCTCTTCTGTCTAC
TNF-α	Reverse	5'- AGGGTCTGGGCCATAGAACT
GAPDH	Forward	5'- GGTTGTCTCCTGCGACTTCA
GAPDH	Reverse	5'- GCCTCTCTTGCTCAGTGTCC
